# Molecular diagnostics of hepatobiliary and pancreatic neoplasias

**DOI:** 10.1007/s00428-024-03744-5

**Published:** 2024-03-01

**Authors:** T. Longerich, A. Stenzinger, P. Schirmacher

**Affiliations:** Institute of Pathology, University Hospital Heidelberg, Im Neuenheimer Feld 224, 69118 Heidelberg, Germany

**Keywords:** Hepatobiliary, Molecular diagnostics, Cancer, Pancreas, Personalized Medicine

## Abstract

Neoplasias of the hepatopancreatobiliary tract are growing in numbers, have the poorest prognosis of all major cancer entities, and thus represent a rising clinical problem. Their molecular diagnostic has dramatically improved, contributing to tumor subtyping, definition of malignancy, and uncovering cases with hereditary predisposition. Most of all, predictive molecular testing allows to identify cases amenable to treatment with the rising number of approved targeted drugs, immune-oncological treatment, and clinical trials. In this review, the current state of molecular testing and its contribution to clinical decision-making are outlined.

**Supplementary Information:**

The online version contains supplementary material available at 10.1007/s00428-024-03744-5.

## Introduction

Neoplasias of the liver, bile ducts, and pancreas belong to the most frequent, clinically most relevant and challenging group of malignancies. In addition, their frequencies are rising, and despite significant improvements in prevention, diagnosis, and treatment, the individual prognosis of most patients is dismal, especially if curative resection cannot be achieved.

Precise diagnosis and in recent years prediction of therapeutic response have gained increasing impact in hepatopancreatobiliary cancer due to more and more differentiated therapeutic approaches and particularly rapidly growing systemic treatment options. Molecular pathology is a cornerstone of these diagnostics and contributes in manifold ways to cancer typing (morpho-molecular subtyping, assessment of malignancy in uncertain constellations, and suspicion of genetic cancer predisposition) and predictive testing to guide systemic therapy.

Molecular testing in hepatobiliary and pancreatic cancers has to reflect and adapt to several challenges: (a) resection material or biopsies (which may be small and/or contain only few tumor cells, especially in pancreatic and bile duct biopsies) and under more rare and specific conditions also liquid testing (blood, bile, cyst fluid) have to be handled, and testing approaches have to be tailored to these specimens. Also, (b) the indication for testing (typing, testing for approved therapies or molecular tumor boards, clinical trials, or even individualized treatment approaches) substantially matters and may soon be extended by the need to test for molecularly based adjuvant and neoadjuvant treatments. Finally, (c) availability of assays, competence, financing, and clinical environment affect the choice of tests and workflows. A peculiarity represents liver biopsy, as it is frequently the prime or even only material available to test for metastatic cancer of extrahepatic (including pancreatic) or unknown primary site.

Recently, the clinical relevance of molecular testing in hepatobiliary cancer has increased. A number of successful clinical trials have led to approvals for molecularly guided systemic therapies. In addition, the complexity of biomarkers has increased from single gene testing via multigene panels addressing all clinically actionable specific genetic alterations to complex marker testing (e.g., tumor mutation burden (TMB), homologous recombination deficiency (HRD), microsatellite instability (MSI)) and even whole-exome sequencing in certain constellations. Complexity of testing, specific tissue issues, and turn-around time represent the triangle of technical challenges molecular pathology is facing, especially in hepatopancreatobiliary cancer. It can be foreseen that this development will not stop and that adequate scaling of specific pathological, biomedical, and bioinformatic expertise, resources, and equipment are required, a challenge which in its completeness may only be addressed by specialized centers and networks.

## Diagnostic molecular pathology

### Tumor subtyping

#### Hepatocellular adenoma

Hepatocellular adenoma (HCA) is a paradigmatic entity for morpho-molecular tumor subtyping. It mainly affects (younger) women without a pre-existing liver disease and is associated with exposure to steroid hormones [1]. In addition, metabolic (e.g., obesity, glycogenosis) and vascular liver diseases may induce HCA formation. HCA subtyping has relevance in terms of potential complications as well as clinical management (Table 1).

HNF1A-inactivated HCAs (H-HCAs) are characterized by prominent fatty change and are negative for fatty acid binding protein 1 (FABP1) by immunohistochemistry [2]. While they show no increased risk of malignant transformation in general, a CTNNB1-independent malignant transformation has been described in patients older than 60 years with lesions > 5 cm in diameter [3]. So far, the transformation risk has not been linked to specific HNF1A mutations. Thus, sequencing of the *HNF1A* gene is currently neither required for diagnosis nor risk assessment regarding malignant transformation. Of note, liver adenomatosis may be observed in patients with HNF1A germline mutations (who may also develop maturity-onset diabetes of the young type 3).

Inflammatory HCA (I-HCA) results from various mutations in genes contributing to activation of IL-6 signaling [1]. It has some peculiar histological features: inflammatory foci, sinusoidal dilatation, and portal tract-like structures harboring ductular proliferations [2]. Positivity of acute phase proteins (e.g., serum amyloid A, C-reactive protein) compared to the surrounding liver tissue can be used as a diagnostic immunomarker. The tumor-associated secretion of acute phase proteins may result in a systemic inflammation, which can be treated by HCA resection [4].

Activating mutations of the *CTNNB* gene characterize a subgroup of HCA, which carries an increased risk of malignant transformation into HCC (so-called ß-catenin-activated HCA, B-HCA) [1, 5–7]. About half of all B-HCA reveal additional features of inflammatory HCA (BI-HCA) [1, 8]. Overall, the frequency of *CTNNB1* mutation in HCA is 10 to 15% [1]. Most mutations affecting exon 3 result in high activity of WNT signaling, while mutations in exons 7 (K335) and 8 (N387) and the S45 mutation in exon 3 lead to weaker pathway activation [6]. The combination of glutamine synthetase (GS) and CD34 immunohistochemistry is able to discriminate these mutations in most cases, but molecular testing is advisable. HCA with classical exon 3 mutations show a diffuse GS expression and increased sinusoidal CD34 expression. Exon 3 S45 mutation is characterized by heterogeneous GS staining associated with a GS-positive but CD34-negative rim, while the central lesion reveals a diffuse capillarization. Exon 7/8 mutations show a similar CD34 staining pattern, but GS positivity is only focal and patchy [9]. Strong activation of WNT signaling resulting from classical exon 3 mutations or S45 allele duplication has been associated with a high risk of malignant transformation [6]. Consequently, molecular testing not only clarifies the precise nature of the *CTNNB* gene mutation but it also provides information about the risk of malignant transformation and is thus predictive in terms of therapeutic decisions (resection of all B-HCA with high WNT-pathway activation).

A rare HCA subtype reveals activation of sonic hedgehog signaling (SH-HCA) due to focal deletions that fuse the promoter of INHBE with GLI1. These tumors occur more frequently in obese patient and have a higher risk of rupture and life-threatening bleeding [1]. Argininosuccinate synthase 1 has been proposed as a diagnostic immunomarker [10]. The very recently described familial adenomatous polyposis (FAP)-HCA occurs in patients with germline mutations of the *APC* gene and shows also activation of the WNT signaling pathway as demonstrated by strong positivity for glutamine synthetase. Thus, this rare subtype shares features with B-HCA, but it does not reveal nuclear beta-catenin accumulation and an increased risk of malignant transformation has not been established for these HCA [11]. Finally, rare HCAs that do not fit in the above-mentioned subtypes are considered unclassified HCA (U-HCA).

#### Hepatocellular carcinoma

Numerous more or less differentiated attempts to subclassify hepatocellular carcinoma (HCC) using molecular genetic testing, expression profiling (RNA- and protein-based), epigenetics, and combinations thereof have been made. These analyses have uncovered molecular mechanisms contributing to different modes of HCC development and have thus provided the basis for further research. None of these approaches has made its way into HCC diagnosis or clinical management of HCC patients, as they have several shortcomings: there are many proposals but no consensus regarding classification schemes and methodology. As only earlier (resectable) tumor stages have been included, it is unclear whether the classification schemes represent molecular tumor typing or staging and to which extent they are valid for progressed HCCs.

**Table 1:** Clinico-pathological features of HCA subtypes

HCA subtype	Molecular alteration	Risk factors	Clinical presentation	Histological features	Diagnostic IHC
H-HCA	*HNF1A* mutations	HNF1A germline mutation	Female, adenomatosis	Steatosis, microadenoma	FABP1 −
I-HCA	*IL6ST*, *STAT3*, *FRK*, *GNAS*, *JAK1* mutations	Obesity, alcohol, glycogenosis	Inflammatory syndrome	Portal tract-like structures, sinusoidal dilatation, inflammatory foci	SAA/CRP +
BI-HCA	*CTNNB1* + *IL6ST*, *STAT3*, *FRK*, *GNAS*, *JAK1* mutations				SAA/CRP + , GS + , (CTNNB1 +)
B-HCA	*CTNNB1* mutations	Androgen exposure, vascular liver disease	Male, malignant transformation (risk: exon 3 > exons 7 and 8)	Cytological atypia	GS + , (CTNNB1 +)
FAP-HCA	*APC* mutations	APC germline mutation	FAP patient		GS + , CTNNB1 −
SH-HCA	*INHBE*-*GLI1* fusion	Obesity	Bleeding	Hemorrhage	ASS1 +
U-HCA	unknown	Oral contraception?			Not defined

− , negative; + , positive; *ASS1*, argininosuccinate synthase 1; *B-HCA*, β-catenin-mutated hepatocellular adenoma; *BI-HCA*, β-catenin-mutated inflammatory hepatocellular adenoma; *BMI*, body mass index; *CRP*, C-reactive protein; *CTNNB1*, beta-catenin gene; *FABP1*, fatty acid binding protein 1; *FRK*, fyn-related kinase; *GLI1*, glioma-associated oncogene 1; *GNAS*, guanine nucleotide binding protein α stimulating; *HCC*, hepatocellular carcinoma; *H-HCA*; HNF1A-mutated hepatocellular adenoma; *HNF1A*, hepatocyte nuclear factor 1A; *IL*, interleukin; *I-HCA*, inflammatory hepatocellular adenoma; *INHBE*, inhibin β E; *JAK*, Janus kinase; *OC*, oral contraception; *SAA*, serum amyloid A; *SH-HCA*, sonic hedgehog hepatocellular adenoma; *STAT*, signal transducer and activator of transcription; *TERT*, telomerase reverse transcriptase; *U-HCA*, unclassified hepatocellular adenoma

It has become apparent that HCC, besides the majority of typical HCCs showing different growth and cytological patterns (may be called HCC, not otherwise specified), contains several specific morpho-molecular subtypes, which show peculiar histological, molecular, clinical, and biological characteristics (Table 2) and whose phenotypes typically remain stable throughout tumor progression. HCC subtyping has been included into the 5th edition of the World Health Organization (WHO) classification. Molecular analyses may support the diagnosis of these subtypes in questionable cases, of, e.g., fibrolamellar, sclerotic, or chromophobe HCCs. Other subtypes, such as lymphocyte-rich and steatotic subtypes, are still less clearly defined and lack consented diagnostically applicable molecular markers.

**Table 2:** WHO subtypes of HCC [24]

Subtype	Frequency	Clinical features	Histology	Molecular features
Steatohepatitic	5–20%	May be associated with steatohepatitis	Fatty change, ballooning, inflammatory foci, Mallory-Denk bodies	IL-6/JAK/STAT activation; low frequency of CTNNB1, TERT, and TP53 mutations
Clear cell	3–7%	Unknown	> 80% of tumor cells with clear cell morphology, mild fatty change is acceptable	Unknown
Macrotrabecular-massive	5%	High-serum AFP, poor prognosis	Macrotrabecular growth in > 50% of tumor, vascular invasion common	TP53 mutation, FGF19 amplification
Scirrhous	4%	May mimic cholangiocarcinoma on imaging	> 50% of tumor showing a dense intratumoral fibrosis	TSC1/2 mutation; activated TGFβ-signaling
Chromophob	3%	Unknown	Tumor cells with chromophobe cytoplasm, mainly bland nuclei, but areas with anaplasia and microcysts	Alternative lengthening of telomeres
Fibrolamellar	1%	Young, no background liver disease	Large oncocytic tumor cells (K7 and CD68 positive) with prominent nucleoli, dense lamellar intratumoral fibrosis	DNAJB1-PRKACA fusion gene
Neutrophil-rich	< 1%	Leukocytosis, CRP and IL-6 elevation, poor prognosis	Prominent infiltration by polymorphic granulocytes, sarcomatoid areas may be seen	G-CSF expression by tumor cells
Lymphocyte-rich	< 1%	Unknown	Lymphocytes > tumor cells	Unknown, not EBV-related

In rare cases, the demonstration of hepatitis B virus-DNA integrations in HCC may establish a causal relation between profession-based infection and HCC development.

#### Cholangiocarcinoma

Intrahepatic cholangiocarcinoma (iCCA) is the second most frequent malignant primary liver tumor entity and needs to be separated from carcinomas of the gallbladder (GBCA) and extrahepatic biliary tree (eCCA). As suggested by animal models, iCCA may (under certain conditions also) develop from hepatocytes [12, 13]. In line with this hypothesis, detection of albumin mRNA expression by albumin in situ hybridization has been proposed to support the diagnosis of small duct (sd)-iCCA [14, 15].

At the histological level, a sd-iCCA composed of non-mucinous, cuboidal cells forming small tubular and ductular structures in a desmoplastic stroma is separated from a large duct (ld) type, which is biologically similar to eCCA and contains mucin-secreting, columnar cancer cells. sd-iCCA shares the etiological risk profile and primary nodular growth pattern with HCC, while ld-iCCA mirrors the etiology and growth pattern of eCCA.

While eCCA and iCCA share some common mutations (e.g., *TP53*, *BRCA1*, *BRCA2*, *PK3CA, KRAS*, *SMAD4*, *ARID1A*, *GNAS*), others are especially typical for small duct-iCCA (*IDH1*, *IDH2, and BAP1* mutations as well as translocations involving *FGFR2*, *NRG1*, *ALK*, *NTRK1-3,* and possibly others; Table 3) and may eventually allow for the identification of iCCA in a cancer of unknown primary (CUP) constellation [16, 17]. This is of direct clinical relevance as a specification of a hepatic adeno-CUP may provide the patient access to several specific guideline- and approval-based targeted and non-targeted therapeutic options superior to standard adeno-CUP chemotherapy.

**Table 3:** Mutational spectrum of eCCA and iCCA (modified according to [58]

Molecular alteration	dCCA/pCCA	iCCA
ARID1A mutation	5–10%	5–15%
BAP1 mutation	0–5%	5–15%
BRAF^V600E^ mutation	0–2%	3–6%
CDKN2A/B mutation	10–20%	10–15%
ELF3 mutation	3–10%	1–2%
ERBB2 mutation	2–5%	2–3%
FGFR2 translocation	0%	15–30%
IDH1/2 mutation	0–3%	10–20%
KRAS/NRAS mutation	20–40%	10–20%
MSI-H	1–3%	1–2%
NRG1 translocation (mostly detected in mucinous adenocarcinoma)	< 1%	< 1%
NTRK translocation	1–3%	1–3%
PBRM1 mutation		10–17%
PRKACA/PRKACB translocation (IPNB-associated)	1–3%	0%
SMAD4 mutation	5–15%	2–10%
TP53 mutation	20–40%	20–30%

There is the first evidence that small duct iCCA, similar to HCC, may contain to a significant extent various different, low-frequency, morpho-molecularly defined subtypes; next to the cholangiolocellular and ductal-plate malformation-like subtypes that are already recognized, the solid-tubulocystic (“cholangioblastic”) subtype with its peculiar morphology, inhibin-positivity, and diagnostic NIPL-NICC1 translocation has recently been defined [18]. Further potential morpho-molecular subtypes have been proposed and await confirmation.

#### Pancreatic cancer

Several attempts to molecularly subclassify pancreatic ductal adenocarcinoma (PDAC) have been carried out based on microarray data obtained from cell lines and PDAC tissue, RNA-sequencing in silico subtraction of transcript data obtained from cells comprising the tumor microenviroment (TME). [16, 17, 19]. Based on these results and additional studies [18–20], there is now some consensus that acknowledges at least two different molecular subtypes with some overlap and inter- and intra-tumor heterogeneities (classic and basal types). Several clinical studies aim at harnessing molecular subtypes as well as other RNA-based signatures to inform efficacy of systemic treatments. Alternatively, assessment of copy number variations (CNVs) and larger chromosomal rearrangements can classify PDAC into four subtypes: “stable,” “locally rearranged,” “scattered,” and “unstable” [20]. These may be exploited clinically in the future, as the unstable subtype is associated with homologous recombination deficiency and there is evidence that CNV-rich tumors tend to display a cold TME. Currently, molecular testing is not required clinically to define or subtype PDAC.

#### Other tumor entities

In other liver tumor entities, molecular testing is rarely required for typing. Rare exceptions may be questionable cases of malignant epithelioid hemangioendothelioma. Here, the demonstration of a WWTR1-CAMTA1 fusion gene (up to 90% of the cases) or the rare YAP1-TFE3 translocation may support the diagnosis in questionable cases [21]. In cases of hepatic adeno-CUP, the pattern of molecular alterations may provide valuable information in regard to the entity. For example, detection of an FGFR2 translocation characterizes the tumor as a sd-iCCA with high certainty and provides the patient with several approved systemic therapeutic options.

### Definition of malignancy

**Fig. 1:**
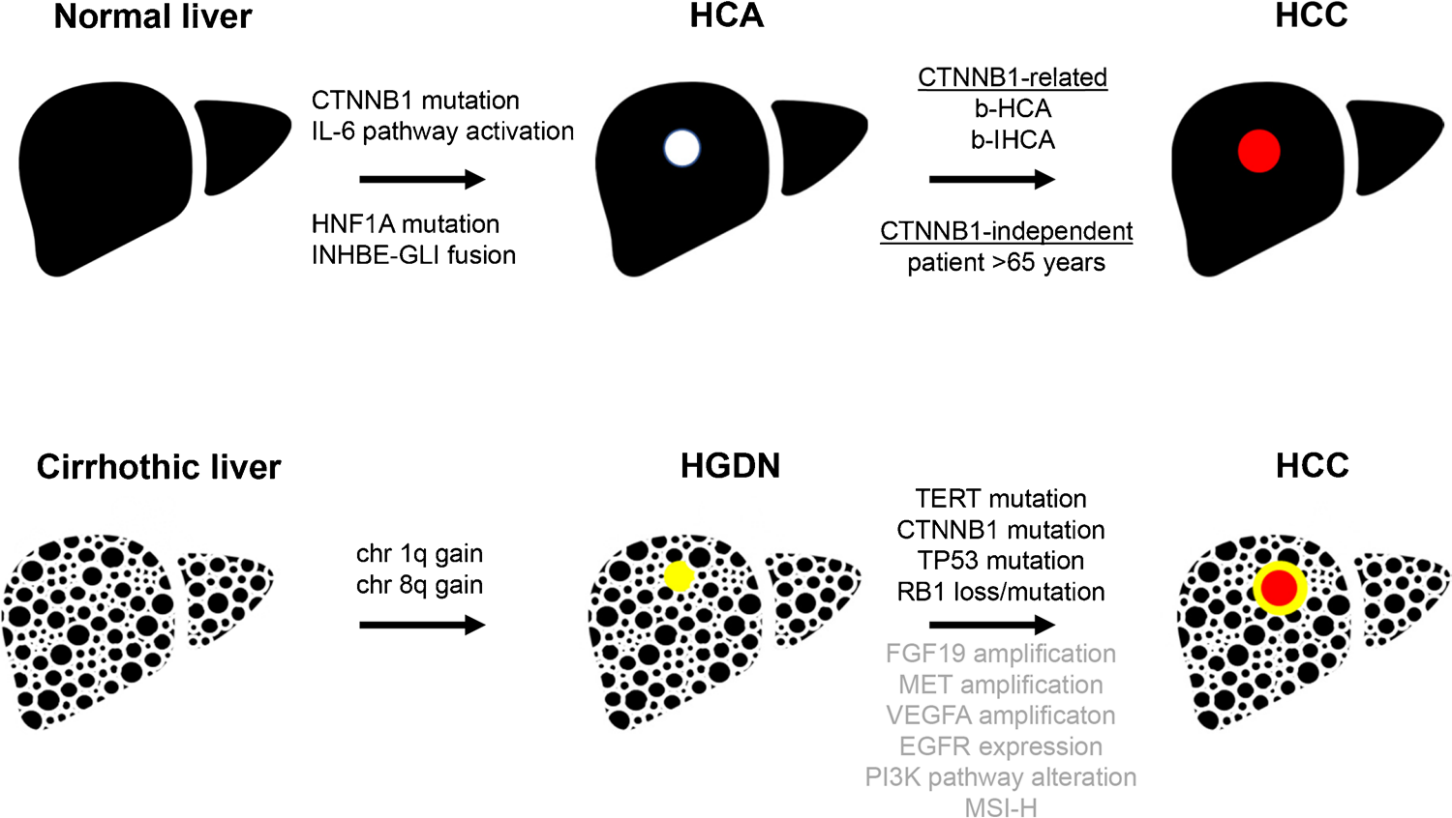
Modes of malignant transformation of HCA are shown in the upper panel. While HCC development on the basis of B-HCA and BI-HCA relies on the presence of the CTNNB1 gene mutation and further acquired alterations, malignant transformation of (mostly HNF1A inactivated) HCA in older patients occurs in setting of a wild-type CTNNB1 gene locus. The development of high-grade dysplastic nodules in cirrhotic livers is shown in the lower panel and is associated with chromosomal gains at 1q or 8q. Frequent mutations involving the transition from dysplastic nodule to HCC are shown in black, while altered candidates potentially useful as drug targets are shown in gray. Abbreviations: *B-HCA*, beta-catenin-activated HCA; *BI-HCA*, beta-catenin-activated inflammatory HCA; *chr*, chromosome; *CTNNB1*, beta-catenin gene; *EGFR*, epidermal growth factor receptor; *FGF19*, fibroblast growth factor 19; *HCA*, hepatocellular adenoma; *HNF1A*, hepatocyte nuclear factor 1A; *GLI1*, GLI family zinc finger 1; *IL-6*, interleukin-6; *INHBE*, inhibin subunit beta E; *MET*, MET proto-oncogene; *MSI-H*, high microsatellite instability; *PI3K*, phosphoinositide-3-kinase; *RB1*, RB transcriptional corepressor 1; *TERT*, telomerase reverse transcriptase; *TP53*, tumor protein P53; *VEGFA*, vascular endothelial growth factor A

Tumor typing includes the reliable distinction of benign or premalignant hepatocellular lesions from early and highly differentiated HCC. One scenario is malignant transformation of B-HCA into HCC and the other the differential diagnosis between premalignant dysplastic nodules and highly differentiated HCC (Fig. 1).

Of note, the histological changes in the surrounding liver tissue as caused by the underlying chronic liver disease together with the so-called matrix diagnosis (e.g., older age, male gender, presence of chronic liver disease, patient origin from high-risk area) may support the diagnosis of HCC vs. HCA or FNH. Since definite histopathological features of malignant transformation (interstitial and vascular invasions) are rarely found in a critical biopsy specimen, next to the demonstration of disturbed trabecular architecture, the diagnosis of well-differentiated HCC may be supported by diffuse capillarization of the sinusoids in HCC as detected by CD34 immunohistology [22]. In addition, an immunohistological marker panel (heat shock protein 70, glypican-3, and GS; Table 4) became the diagnostic standard for the molecular adjunct diagnosis for malignancy in highly differentiated hepatocellular tumors. It provides a high sensitivity (~ 70%) and a near perfect specificity for the detection of malignancy in independent studies [23]. Moreover, detection of mutations in the telomerase reverse transcriptase promoter may be helpful for the identification of malignant transformation in highly differentiated hepatocellular tumors, namely, HCA and dysplastic nodules vs HCC [7]. It employs the fact that hTERT promoter mutations significantly increase in frequency from HCA (0%) to “borderline” cases (17%) to HCC derived from HCA (56%) [7] and from dysplastic nodules (6–19%) to early and highly differentiated HCC (43–61%) [24]. It has to be noted that the detection of hTERT-promoter mutations requires specific DNA-PCR-based assays and cannot be detected by standard panel-based assays or WES.

**Table 4:** Diagnostic performance of HCC marker panel (modified according to [59]

Phenotype	HGDN	G1-HCC	Sensitivity	Specificity	PPV	NPV
3 marker						
HSP70 + /GPC3 + /GS +	0%	44%	44%	100%	100%	55%
2 markers positive	0%	72%	72%	100%	100%	71%
1 marker positive	27%	91%	91%	73%	83%	84%
2 marker						
HSP70 + /GS +	0%	53%	53%	100%	100%	59%
HSP70 + /GPC3 +	0%	59%	59%	100%	100%	63%
GPC3 + /GS +	0%	47%	47%	100%	100%	56%
1 marker						
HSP70 +	5%	78%	78%	95%	96%	75%
GPC3 +	9%	69%	69%	91%	92%	67%
GS +	14%	59%	59%	86%	86%	59%

*PPV*, positive predictive value; *NPV*, negative predictive value; *G1-HCC*, well-differentiated HCC

In about 85–90% of the cases, PDAC may develop from different premalignant precursors: pancreatic intraepithelial neoplasia (PanIN) progresses from low- to high-grade lesions accumulating genetic alterations (e.g., mutations in KRAS, SMAD4, TP53) [25, 26]. Furthermore, intraductal papillary mucinous neoplasms (IPMNs) may progress from low-grade to high-grade dysplasia to PDAC, and more rarely mucinous cystic neoplasms (MCNs) may malignantly transform. KRAS mutations can be observed early in neoplastic development (i.e., even in low-grade PanIN and IPMN) consequently increasing to more than 90% of PDAC carrying activating KRAS mutations as major driver event. While diagnosis of malignant transformation in pancreatic carcinogenesis resides solely on histology not requiring any molecular testing, molecular testing of aspiration fluid may help to clarify the nature of cystic pancreatic lesions [27, 28].

### Genetic cancer syndromes and genetic tumor predisposition

Affection of the liver and biliary tree in genetic cancer syndromes is very rare. The vast majority of cases of HCC with a genetic background are due to hereditary metabolic diseases (e.g., genetic iron storage diseases, hereditary tyrosinemia type I (rare), Wilson’s disease (rare)). In these cases, severe hepatic disease manifestation provides the soil for HCC and iCCA development. Consequently, prevention of liver affection abolishes the risk of tumor development. The reason for the relative protection in regard to hepatic involvement in genetic tumor predisposition syndromes is unknown. Thus, there is no indication to test for genetic cancer syndromes in HCC and CCA. Genetic predisposition by respective germline mutations is likely in hepatic adenomatosis (> 10 HCA in a patient) or when histology or immunohistology detect multiple comparable microlesions in the non-tumorous parenchyma of a HCA-resection specimen. Angiomyolipoma (AML) has been linked to the tuberous sclerosis complex, but this correlation is much lower in hepatic AML when compared to renal AML. Rarely, hereditary cases of pancreatic cancer have been observed in association with Peutz-Jeghers syndrome (STK11), hereditary pancreatitis (PRSS1, SPINK1, CFTR), familial melanoma (CDKN2A, CDK4, BAP1), Lynch syndrome (MLH1, MSH2, MSH6, PMS2), hereditary breast and ovarian cancer syndrome (BRCA2, BRCA1, PALB2), Li-Fraumeni syndrome (TP53), FAP (APC), ataxia telangiectasia, and polymerase proofreading-associated polyposis (POLE, POLD1) [29, 30].

## Predictive molecular pathology

### Hepatocellular carcinoma

Despite the presence of potentially targetable molecular alterations, no entity-specific targeted treatment has reached approval in HCC, so far. Clinical trials addressing MET overexpressing or RAS-mutated HCCs have failed to show overall survival benefit, likely due to shortcomings in testing strategy or drug efficacy. Furthermore, the lack of biopsies in HCC has severely limited predictive testing in HCC trials, and trial-associated molecular analyses and several trials employing pathway-directed drugs have not relied on predictive testing [31]. The frequency of alterations providing entity-independent access to specific systemic therapy, such as NTRK translocations, homologous recombination deficiency, or MMR deficiency, is exceedingly rare and does not justify regular diagnostic testing. Current first- and second-line systemic treatment approaches do not require molecular testing despite the growing evidence that treatment response depends on the molecular characteristics of the HCC. “Immuno-hot” HCCs are far more likely to respond to immune-oncological treatment [32, 33], while CTNNB1-mutated HCCs are rather “immuno-cold” and seem to be better responders to TKI. Lenvatinib appears to act on FGFR-activated HCC, and resistance appears to involve compensatory activation of the EGFR-pathway [34]; furthermore, negative and positive molecular predictors of sorafenib response are likely to exist [35, 36]. Nevertheless, current predictive molecular testing in HCC is largely restricted to broad testing in molecular tumor boards and individual off-label attempts (Supplementary Material 1).

### Cholangiocarcinoma

Most CCA patients are diagnosed with advanced disease. Combined cisplatin and gemcitabine treatment improved the median overall survival and became the standard first-line systemic therapy for more than a decade [37]. Data of the TOPAZ-1 trial showed improved overall and progression-free survival in patients with advanced biliary tract cancer, when the PD-L1 inhibitor durvalumab was added to this therapy regimen [38].

Typically, second-line therapy on molecular alterations may affect treatment decisions significantly. Of the common genetic alterations described above, *IDH1* and *BRAF*^*V600E*^ mutations as well as *FGFR2* fusions have gained primary clinical attention. In the ClarIDHy study, the IDH1 inhibitor ivosidenib demonstrated a clinical benefit in previously treated, advanced IDH1-mutant cholangiocarcinoma [39] and has gained approval for second-line treatment. In addition, dual BRAF and MEK inhibition showed promising activity in patients with *BRAF*^*V600*^-mutated biliary tract cancer in a phase 2 study.

Another recurrent molecular feature of iCCA is the presence of principally targetable gene fusions [40]. In particular, *FGFR2* gene fusions show a high prevalence and have become an attractive target. Initially, the FIGHT-202 study demonstrated for the first time that a selective, oral FGFR inhibitor resulted in an objective response in previously treated CCA patients with detectable FGFR2 gene rearrangements [41], a finding leading to the approval of pemigatinib monotherapy for the treatment of adults with FGFR2 fusion-positive CCA that have progressed after at least one prior line of systemic therapy. In addition, infigratinib has been approved for the treatment of advanced, refractory CCA. Both compounds are ATP-competitive, binding reversibly to the ATP-binding pocket in the FGFR kinase domain, inhibitors universally resulting in acquired resistance mutations [42]. Next-generation inhibitors covalently binding FGFR also led to measurable clinical benefit [43]. Thus, there is a continuously evolving landscape of clinically relevant FGFR inhibitors.

Other gene rearrangements that are amendable for efficient drug targeting include fusions involving the *NRG1* and *NTRK* genes [44, 45]. Although inactivating mutations of genes involved in DNA repair (e.g., MLH1, MSH2, MSH6, PMS2, POLE) may be rarely (~ 1% frequency of pathogenic or likely pathogenic variants) detected in all types of cholangiocarcinoma, they represent a valuable target for off-label treatment immune checkpoint blockade [46].

Meanwhile, at least in dedicated centers, molecular pathological analysis is recommended for every patient with advanced iCCA, which should at least cover the whole spectrum of FGFR2 fusions, IDH1 and BRAF mutations, and NTRK fusions and microsatellite instability. However, more than 50% of iCCA contain potentially druggable alterations, and we recently demonstrated that molecular profiling using large DNA and RNA panels can improve patients’ survival in clinical practice [47, 48]. Molecular alterations that were successfully addressed in addition to the targets detailed above included BAP1, BRCA1, IDH2, and PIK3CA mutations, ERBB2 amplification, and MET and NRG1 fusions [48] (Supplementary Material 1).

### Pancreatic cancer

While specific approvals for targeted therapy are lacking and entity-agnostic approvals face extremely low frequencies of respective alterations in PDAC, some signs of improvement are appearing. About 90% of pancreatic ductal adenocarcinoma are driven by KRAS-mutations and have escaped targeted therapeutic attempts, so far. But the advent of allele-specific (G12C in approximately 1.5% of PDAC, G12D in > 40%) and allele-agnostic small molecule inhibitors of KRAS may influence the treatment landscape. The remaining approximately 10% of PDACs, which display wild-type KRAS, may carry gene fusions involving various drivers (e.g., NRG1, BRAF, ALK, NTRK1-3), in principle amenable to treatment approaches [49, 50]. Treatment with NTRK inhibitors is categorized as IC according to ESMO-ESCAT. These PDAC cases, which are clinically associated with younger onset (< 50 years) require specific attention: KRAS wild-type PDAC should be analyzed by appropriate assays to interrogate genetic translocations leading to potentially druggable gene fusions (e.g., break-apart fluorescence in situ hybridization, RNA-based targeted NGS). This approach has also been endorsed by ESMO guidelines (Supplementary Material 1) [51]. Approximately 5–7% of PDAC harbor mutations in HRR (homologous recombination repair)-encoding genes. These are mostly germline events often followed by a second somatic hit, both of which can be identified in the tumor tissue. These tumors exhibit an HRD (homologous recombination deficiency) phenotype which renders the tumor sensitive to PARP inhibitors or platinum-based agents. PARP inhibitors were shown to prolong progression-free survival in cases of pathogenic/likely pathogenic variants in BRCA1 and BRCA2 but failed to show overall survival benefits [52]. Nevertheless, current guidelines recommend testing BRCA1/2 status [51] (ESMO-ESCAT category: IA). Very few cases of PDAC (approx. 0.5–1.0%) are associated with deficient mismatch repair (dMMR) [53, 54] and trial data show a moderate to good response to checkpoint inhibitor blockade. Given these data as well as the limited therapeutic options, dMMR testing by immunohistochemistry is recommended (ESMO-ESCAT category: IC). Complementary PCR-based assays and NGS may support dMMR/MSI-H profiling [55].

### Cancers of unknown primary (CUP)

Adeno-CUP of the liver is a frequent, clinically relevant constellation that requires specific consideration. Even if molecular testing may not narrow in on the responsible entity, comprehensive predictive testing can be of value as it may offer patients specific therapeutic options beyond the standard non-targeted chemotherapy (Supplementary Material 1). Recent trial data addressing the clinical utility of molecularly guided therapy versus standard platinum-based chemotherapy in patients with unfavorable non-squamous CUP demonstrated significantly improved HR and response rates for patients which received a therapy based on predictive molecular biomarkers. Accordingly, the current ESMO guideline [56] strongly recommends molecular pathology-guided testing in the diagnostic work-up of CUP patients.

## Perspective

Numerous different developments can be foreseen, with significant implications for molecular pathology diagnostics. The number of approved drugs that may require predictive testing will increase further. Novel clinical settings that will significantly increase molecular testing include molecularly targeted drug-antibody conjugates, the extension of targeted drugs, and the respective molecular testing to the adjuvant (as it has already happened in breast and lung cancer) and neoadjuvant setting as well as the integration of (mutation-based) neo-antigen targeted immuno-oncological treatment. Morpho-molecular subtyping in HCC is far from being complete, and it has just begun in iCCA; thus, new subtypes requiring respective molecular testing in suitable diagnostic constellations can be expected.

Molecular pathology diagnostics will have to respond to these challenges in an adaptive manner, taking the indication and the material, resources, and workflow constellation into account. Considering the complex nature and diversity of entities, indications, and markers, nucleic acid–based testing will more and more develop towards “one-size-fits-all/many” approaches and “one-stop-shop-workflows” to meet the time, resources, and material constraints.

Importantly, successful implementation of personalized oncology approaches and thus advanced molecular testing is critically processing time-dependent. This includes the time required for molecular testing, recommendation of a personalized therapy or clinical trial, and access to and financing of potentially suggested off-label therapies. Dedicated clinical infrastructures, like the Centers for Personalized Medicine in the Southwest of Germany, may provide a comprehensive framework (broad molecular testing and molecular tumor boards) for implementation of precision oncology approaches and may help to reduce dropout rates in molecular testing and treatment in progressed tumor stages. [57].

### Supplementary Information

Below is the link to the electronic supplementary material.

Supplementary file1 (DOCX 34.1 KB)
