# Supplementary material for: Molecular diagnostics of hepatobiliary and pancreatic neoplasias
Source: Virchows Arch. 2024 Mar 1;484(2):263–72. doi: 10.1007/s00428-024-03744-5 (PMC10948571; doi:10.1007/s00428-024-03744-5)
Supplement: Supplementary file 1 — Supplementary file1 (DOCX 34.1 KB) [file 428_2024_3744_MOESM1_ESM.docx]

**Molecular Diagnostics of Hepatobiliary and Pancreatic Neoplasias**

**T. Longerich, A. Stenzinger, P. Schirmacher**

Institute of Pathology, University Hospital Heidelberg

Im Neuenheimer Feld 224, 69118 Heidelberg, Germany

Corresponding author: Peter Schirmacher; Peter.schirmacher@med.uni-heidelberg.de

**Supplementary Table 1**: Molecular targets in advanced carcinomas of liver and pancreatobiliary system.

| **Gene** | **entity** | **Class** | **Alteration** | **Drug class** | **ESCAT** | **NCT variant classification** | **Reference** |
| --- | --- | --- | --- | --- | --- | --- | --- |
| ALK | iCCA | Oncogene | fusion | ALK inhibitor | IIIA | m2A | approved for ALK-positive lung cancer |
| ARID1A | iCCA | TSG | mutation | PARP inhibitor | IVA | m2C | PMID: 31764178 |
| ARID2 | iCCA | TSG | mutation, deletion | Immune checkpoint inhibitor | IIIA | m2B | PMID: 32321774 |
| ATM | iCCA | TSG | mutation | PARP inhibitor | IIB |  | NCT04042831 or analogous to |
| BAP1 | iCCA | TSG | mutation | PARP inhibitor, EZH2 inhibitor | IIB | M1c-M3 / m2A | NCT03207347 or analogous to |
| BRAF | iCCA | Oncogene | V600E / other activating mutation / fusion | BRAF inhibitor plus MEK inhibitor | IB | m1A / m2c | Subbiah et al., Lancet Oncol 2020 |
| BRCA1 | iCCA | TSG | mutation | PARP inhibitor | IIB / IIIA | NA / m2A | NCT04042831 or analogous to / POLO Trial (PMID: 31157963) |
| BRCA2 | iCCA | TSG | mutation | PARP inhibitor | IIB / IIIA | NA / m2A | NCT04042831 or analogous to / POLO Trial (PMID: 31157963) |
| CDK4 | iCCA | Oncogene | amplification | CDK4/6 inhibitor | IVA / IIB | m3 / NA | PMID: 30084835 / NCT03310879 or analogous to |
| CDKN2A | iCCA | TSG | deficiency | Aurora kinase inhibitor | IIB | NA | NCT02478320 or analogous to |
| CHEK2 | iCCA | TSG | deficiency | PARP inhibitor / CHECK inhibitor | IIB | NA | NCT04042831 or analogous to / NCT02873975 or analogous to |
| ERBB2 | iCCA | Oncogene | amplification | HER2 inhibitor | IIIA | m1C | PMID: 26022204 |
| FANCE | iCCA | TSG | mutation | PARP inhibitor | IIB |  | NCT04042831 or analogous to |
| FGFR2 | iCCA | Oncogene | rearrangement | Pemigatinib | IB | m1A | Approved |
| FGFR2 | iCCA | Oncogene | mutation | FGFR inhibitor | IIb |  | NCT03230318 or analogous to |
| FGFR3 | iCCA | Oncogene | alteration | FGFR inhibitor | IIIA | m2A | Approved for other entities |
| IDH1 | iCCA | Oncogene | mutation | Ivosidenib | IA | m1A | approved |
| IDH2 | iCCA | Oncogene | mutation | Enasidenib |  | m2a | FDA approved for AML (IDH2) |
| MET | iCCA | Oncogene | mutation, fusion | Crizotinib | IV | m1C | PMID: 32897609 |
| MLH1 | iCCA | TSG | inactivation | Pembrolizumab | IIA | m1B/Z (FDA) |  |
| NF1 | iCCA | TSG | mutation | Ipatasertib + Atezolizumab) / Everolimus / Selumetinib |  | m2b / m1c-2b / m2b | NCT04551521 or analogous to / analogous to NCT02352844 / analogous to NCT02644512 |
| NF2 | iCCA | TSG |  | Everolimus |  | m1c-2b | analogous to NCT02352844 |
| NRAS | iCCA | Oncogene | mutation | Binimetinib | IIB |  | NCT04439344 or analogous to |
| NRG1 | iCCA | Oncogene | fusion | Afatinib / Erlotinib/Pertuzumab | IVA | m1c / m2c | PMID: 28950338 / PMID: 29802158 |
| PALB2 | iCCA | TSG | mutation | First line Platinum based, Second line PARP-Inhibitor | IIB |  | NCT04042831 or analogous to |
| PBRM1 | iCCA | TSG | mutation | PARPi, ATR Inhibitor | IVA | m3 | PMID: 33888468 |
| PIK3CA | iCCA | Oncogene | mutation | Alpelisib / Taselisib / Everolimus, Temsirolimus | IIIA / IIB | m2a / m2a /m1c | analogous to SOLAR-1 trial (w/o anti-hormonal treatment) / NCT04439175 / PMID: 28694672 |
| PIK3R1 | iCCA | TSG | mutation | AKT Inhibitor | IVA | m3 | PMID:23619167 |
| PMS2 | iCCA | TSG | inactivation | Pembrolizumab | IIA | m1b/Z (FDA) |  |
| POLE | iCCA | TSG | mutation | Checkpoint Inhibitor | IIB |  | NCT03428802 |
| PTEN | iCCA | TSG | deletion, mutation | Pik3/AKT/mTOR Inhibition, z.B. off Label Everolimus / CRAFT Studie-Arm: Ipatasertib + Atezolizumab) / PI3Kbeta Inhibitor AZD8186 | IIIA / IIB | m2a-b | PMID:23582881 / NCT04551521 / NCT03218826 |
| RAC1 | iCCA | Oncogene | mutation | simvastatin / pamidronate | IV | m3 | PMID: 21334995 / PMID: 31611937 |
| SMARCA4 | iCCA | TSG | mutation, deletion | tazemetostat (EZH2 inhibitor) | IIB | m2A | approved for epithelioid sarcoma, follicular lymphoma, NCT04241835 or analogous to |
| VHL | iCCA | TSG | mutation | Sunitinib | IVA | m1C | PMID: 32552305 |

| druggable targets |
| --- |
| potentially druggable targets |
| eventually druggable targets |
